# Parallel Mechanisms for Visual Search in Zebrafish

**DOI:** 10.1371/journal.pone.0111540

**Published:** 2014-10-29

**Authors:** Michael J. Proulx, Matthew O. Parker, Yasser Tahir, Caroline H. Brennan

**Affiliations:** 1 Crossmodal Cognition Lab, Department of Psychology, University of Bath, Bath, United Kingdom; 2 School of Biological and Chemical Sciences, Queen Mary University of London, London, United Kingdom; University Zürich, Switzerland

## Abstract

Parallel visual search mechanisms have been reported previously only in mammals and birds, and not animals lacking an expanded telencephalon such as bees. Here we report the first evidence for parallel visual search in fish using a choice task where the fish had to find a target amongst an increasing number of distractors. Following two-choice discrimination training, zebrafish were presented with the original stimulus within an increasing array of distractor stimuli. We found that zebrafish exhibit no significant change in accuracy and approach latency as the number of distractors increased, providing evidence of parallel processing. This evidence challenges theories of vertebrate neural architecture and the importance of an expanded telencephalon for the evolution of executive function.

## Introduction

Prioritising sensory information is a fundamental problem for all animals. Even the relatively large human brain [1] can process only a fraction of the potential input [2]. The most efficient way to process information is to process multiple fragments of information in parallel, such as evaluating several objects in visual search to detect a target [3], [4]. A critical marker of parallel processing is that time and accuracy to locate a target does not increase when the number of items to evaluate is increased. Although prior work has demonstrated contrast detection, or saliency, mechanisms in numerous species [5], there is no direct evidence for parallel processing in visual search by fish.

Given the adaptive benefits of being able to find a target mate, predator, or prey efficiently, parallel visual search should be common. Surprisingly, parallel visual search has thus far only been discovered in primates [4], rats [6], and pigeons [7]. Such visual processing abilities are thus often thought to be supported only by neural circuits in cortical areas [8]. Consistent with a cortex-dependent mechanism, animals lacking an expanded telencephalon like honeybees [9] use serial visual search mechanisms and cannot assess multiple items in parallel.

It has been suggested that birds are able to match primates in cognitive sophistication as a result of convergent evolution [10], perhaps through adaptation of shared rudimentary circuits. If so, then one would predict that other vertebrates, such as fish, would be able to perform parallel search despite the lack of an expanded telencephalon and ‘high-level’ cortex. Primate studies have considered the role of the superior colliculus [11], a midbrain area that might be the homologue of the optic tectum in zebrafish; however the superior colliculus is often dismissed, with most favouring either parietal cortex [12] or primary visual cortex [13] for parallel search.

Here we assessed whether the zebrafish might utilize parallel mechanisms for visual search in the absence of an expanded telencephalon [14]. Cholinergic-mediated attentional mechanisms [8] are thought to mediate parallel processing, and cholinergic neural circuits are present in zebrafish [15], with similar connectivity to that seen in mammals [14]. Surprisingly, although the zebrafish relies on vision extensively, most studies of zebrafish examine low-level oculomotor reflexes rather than higher-level visual behaviours [16]. Recently, however, we reported that zebrafish are capable of acquiring and maintaining an attentional set for colour in a discrimination task [17]. Zebrafish can also carry out visual feature binding for social behaviours like shoaling [18]. Building on these observations we hypothesized that more complex behaviours dependent on the optic tectum may be possible even in the absence of the functional architecture of the mammalian visual, parietal and frontal cortices. These prior studies in zebrafish assessed neither how much information could be processed nor reported whether the information processing load influenced the latency of processing and response. Here we addressed the issue of processing load and latency for the first time by requiring fish to learn an abstract visual search task that would allow for an assessment of whether such processing was serial or parallel.

## Methods

### Ethics Statement

All animal work was carried following approval from the Queen Mary Research Ethics Committee, and under licence from the Animals (Scientific Procedures) Act 1986. Care was taken to minimize the numbers of animals used in this experiment in accordance with the ARRIVE guidelines (http://www.nc3rs.org.uk/page.asp?id=1357; see Checklist S1). Specifically, we examined data from previous pilot studies and studies with other species to carry out a power calculation and assess the minimum number of animals necessary for the expected effect size with power of 0.8.

### Subjects

**Figure 1:**
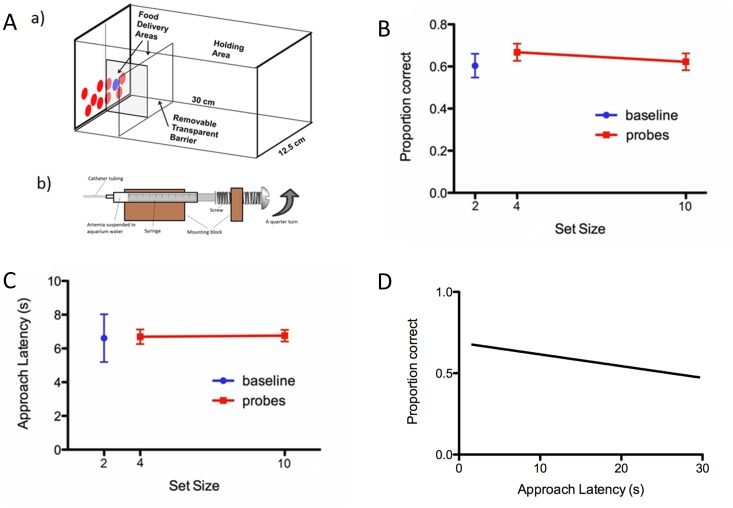
Zebrafish performance on 2-choice discrimination. A) Fish were trained in a glass tank (a), within a light-and-sound attenuating box. The divider was raised at the start of each trial, allowing the fish access to the discriminanda. Food reinforcement (artemia suspended in aquarium water) was delivered via a custom-made device (b; adapted, with permission, from 17). B) Percentage of correct responses as a function of set-size (error bars represent standard error). C) Approach latency response times as a function of set-size (error bars represent standard error). Accuracy and response time were unaffected by discrimination set-size, suggestive of parallel search. D) Speed accuracy trade-off function. There was no correlation between accuracy (*y* axis) and response latency (*x* axis), suggesting that fish did not trade-off speed for accuracy here, further suggesting parallel processing was occurring during discrimination performance in the zebrafish (*r* = −0.06).

The subjects were 11 adult zebrafish (∼1 year old at start of testing, AB wild-type strain, n = 5 male), bred and reared in a UK Home Office licensed aquarium facility. The fish were kept at 28°C on a 14 hr:10 hr light:dark cycle (lights on 9 am) and housed in aquarium water (de-ionized water with added marine salts). The test tanks temperature was also approximately 28°C (27°C ±2). All tanks were fitted with air-lines and regularly monitored for water quality. Tank water was changed weekly. The experimental unit was fish nested in tank (i.e., tank was added to statistical models as a random effect). Fish were fed only during behavioural testing, except at weekends. During this time, fish were fed three times each a day; twice with brine shrimp (morning and late afternoon) and a mid-day feed of flake food. A session comprised 20 discrete trials so that the maximum number of brine shrimp was 200 µl (delivered with the mechanism shown in Figure 1B), with supplemental food provided after testing if the fish received fewer than five rewards. Following testing, the fish were returned to our breeding stock.

### Apparatus

Testing was performed in a transparent glass tank (Figure 1A) with dimensions (L × W × H) 30 cm × 12.5 cm × 20 cm. The areas where the stimuli were presented were (L × W) 10 cm x 6 cm. Water level was maintained at 15 cm. The testing tank was located within a light-and sound-attenuating box, and the experimenter controlled the barrier and food delivery. The background was clear, but in a darkened environment. Habitution time was unnecessary due to the fish having prior experimental experience. A trial started with the lifting of the transparent gate and presentation of the visual stimuli on a 15 inch flat screen monitor (60 Hz) adjacent to one wall of the tank. A PowerPoint presentation was used to generate images that were presented on the computer screen in a pseudo-random order with respect to both side and location. Randomized partial counterbalancing was employed by random selection of as many sequences as fish so as to minimize order effects with each fish subject to a different order. Stimulus presentation with computer monitors has been established in other areas of zebrafish behaviour, such as anti-predator [19] and social [20] behaviour. The barrier was lifted via a pulley system outside of the box. The pulley was lowered and the gate was weighted, and thus lowered. The zebrafish were first trained in a baseline colour discrimination task, with blue as the target and red as the distractor. We chose a non-optimal colour, blue, to insure that any behaviours observed were not reflexive, but required the target feature to be learned because red is associated with feeding and preferred [21], and the target was less salient than the distractors (the background was dark blue, making the red more salient than blue).

### Procedure

#### Baseline trials

On each baseline trial one red disk and one light blue disk appeared on the dark background, one on each side of a central barrier to provide a two alternative forced choice task. The side and location of the blue target disk was pseudo-randomized from trial to trial and appeared equally on both sides (10 trials each). The fish were deemed ready to be tested on increasing set sizes when it made six consecutive correct choices in a 20-trial session (the probability of this occurring by chance is 0.015).

#### Increased set size trials

The probe testing sessions were carried out in the same manner, except the number of stimuli shown varied to test the efficiency of target selection, with a total set size of 4 or 10 disks. One was always the target disk, and the number of items and the location of the target were presented with randomized partial counterbalancing with 10 trials at each set size interspersed with the target appearing in each of the two locations presented each day.

Each trial began when the stimuli appeared and the barrier lifted. Response time, or the approach latency, was defined from the time the time the stimuli appeared and the barrier was raised for the fish to see and approach the stimuli, until the first entrance to the feeding area (one screen or the other), at which point the time was stopped as the barrier went down to keep the fish in the feeding area near the chosen stimuli. Once the fish approached the screens with the colour disks the barrier was lowered, and the fish was restricted to the food delivery area for 10 s, with the food reinforcer delivered if the fish had made the correct selection. During this time, the stimuli remained on the screen to improve learning performance [17]. Reinforcers (brine shrimp suspended in aquarium treated water) were delivered via a syringe with catheter tubing (d = 1 mm; Figure 1B) with a bolt and plunger that facilitated the delivery of ∼10 µl of food by turning the screw one quarter-turn [17]. The fish was observed via a live video feed, and the accuracy and latency of response was recorded.

The data were fitted to linear mixed effects models and no response times were trimmed due to the lack of comparable data to allow unambiguous identification of trials as outliers [22]. In all models, ‘tank’ was added as a random effect to account for inter-tank differences (fish were pair-housed). Distribution of studentized residuals was checked for normality where appropriate (for any Gaussian models) following model fitting to ensure the assumptions were met.

## Results

All animals were included in the analysis. The fish underwent prior training colour discrimination [17] and then acquired the target identity for this task within six days, as the fish were required to achieve six correct choices in a row for the learning criterion to be passed (*p*<0.015). They were then tested for the baseline performance for discriminating the target from one distractor (see set size 2 in Figure 1B).

To test the hypothesis that zebrafish use parallel search mechanisms, the fish underwent visual search testing with larger set sizes of 4 or 10 items. The approach latency response times did not increase as the number of distractors increased (*F*_1,353_ = 0.002, *p* = 0.95); the rate of 0.0257 s per item is statistically indistinguishable from zero (INT = 0.642; Figure 1C). The accuracy of response did not decrease as the number of distractors increased (*z* = 0.78, *p* = 0.43), with a slope of −0.0038 relating accuracy to set size (INT = 6.613; Figure 1B). In addition the accuracy of response was greater than chance performance (50%) for both the 2-item condition (*t* (4) = 4.07, *p* = .015; 95% CI [55.3%, 77.9%]) and the 6-item condition (*t* (4) = 3.05, *p* = .038; 95% CI [51.1%, 73.3%]). Furthermore there was no correlation between accuracy and response time (Figure 1D; there were neither main nor interaction effects of latency and accuracy as a function of set size; *z* = −1.35, *p* = 0.176), suggesting that there was not a speed-accuracy trade-off contaminating the results. In fact, there was a non-significant trend in the data suggesting that slower response times were less accurate, further buttressing the lack of a correlation between accuracy and response time.

## Discussion

Models of visual search [4] make explicit the mechanisms that guide attention for finding a target amongst multiple distractors by evaluating the efficiency or rate of visual search as the number of distractors is increased. Given that serial search mechanisms are revealed by a positive slope (generally greater than 10 ms per item in the human literature [4]), a slope of zero suggests that parallel mechanisms of target detection are implicated here. The parallel processing exhibited by the zebrafish here is likely of a limited capacity. Pop-out would suggest extremely rapid responses, as seen in humans for a task such as this. As these responses by the fish are slower, they are not pop-out yet still parallel as the response times do not increase with the increase in the number of items in the display. This perspective is extended by theoretical work suggesting that limited capacity parallel search is the norm for humans in most tasks [23]. It is interesting to note a study examining the development of visual search abilities found that, in a similar task to that shown to the fish here, the response time functions relating search time to the number of items for children and adults were flat (a slope of zero). However it was also found that the intercept, and thus average response time, was much slower – six times as slow – in children than adults [24].

When considered in tandem with recent work on attentional sets in zebrafish [17], these findings imply that the fish were using an attentional set for the specific target colour, blue, rather than just detecting a unique item independent of its colour value [25]. Future work examining whether the fish were relying on bottom-up mechanisms to detect a unique item or top-down mechanisms set for a particular colour would be of particular interest [25]. The fish no evidence for a speed-accuracy trade-off, unlike what has been found in visual search by bees [26], [27]. What might happen if the number of targets were further doubled? These findings may inform models zebrafish shoaling [28], [29], to the extent that collective behaviour requires the perception and represention of the number of conspecifics within a spatial region [30].

These results imply that the optic tectum in zebrafish might be sufficient to process the multiple items in parallel as implied by the maintenance of accuracy and response time in the face of increasing distractors during visual search. Such a result is consistent with the primacy of the superior colliculus for visual search in mammals [11], or perhaps challenges theories of vertebrate neural architecture and the evolution of executive function by suggesting zebrafish have a homologue of lateral prefrontal cortex [31], such as the pallium in adult [32], or sub-pallium in juvenile zebrafish [33]. Alternatively, it is not the existence of frontal or other cortices that is important, but the existence of common neurotransmitter pathways and circuitry. Further work that requires explicitly either top-down or bottom-up processing [25] will provide an opportunity to explore the use of these attentional mechanisms in the zebrafish model, such as whether the exact same neurotransmitter pathways are involved [8]. Given the transparent nature of the larval forms and adult *Casper* mutants [34], and the ease of forward genetic screens in zebrafish, the results reported here represent the first step towards pursuing the physiology, anatomy, genetics and development of a tractable neural circuit for the processing of visual priority.

## Supporting Information

Checklist S1 **ARRIVE guidelines checklist.**
(DOC)
